# Simultaneous Separation of Actinium and Radium Isotopes from a Proton Irradiated Thorium Matrix

**DOI:** 10.1038/s41598-017-08506-9

**Published:** 2017-08-15

**Authors:** Tara Mastren, Valery Radchenko, Allison Owens, Roy Copping, Rose Boll, Justin R. Griswold, Saed Mirzadeh, Lance E. Wyant, Mark Brugh, Jonathan W. Engle, Francois M. Nortier, Eva R. Birnbaum, Kevin D. John, Michael E. Fassbender

**Affiliations:** 1 Chemistry Division, Los Alamos National Laboratory, P.O. Box 1663, Los Alamos, NM 87545 USA; 2 Nuclear Security and Isotope Technology Division, Oak Ridge National Laboratory, Oak Ridge, TN 37831 USA; 3 TRIUMF, Vancouver, BC V6T 2A3 Canada; 4 University of Wisconsin, Madison, WI 53706 USA

## Abstract

A new method has been developed for the isolation of ^223,224,225^Ra, in high yield and purity, from a proton irradiated ^232^Th matrix. Herein we report an all-aqueous process using multiple solid-supported adsorption steps including a citrate chelation method developed to remove >99.9% of the barium contaminants by activity from the final radium product. A procedure involving the use of three columns in succession was developed, and the separation of ^223,224,225^Ra from the thorium matrix was obtained with an overall recovery yield of 91 ± 3%, average radiochemical purity of 99.9%, and production yields that correspond to physical yields based on previously measured excitation functions.

## Introduction

Radium-223 chloride (t_1/2_ 11.4d) is available as a U.S. FDA approved pharmaceutical for the treatment of bone metastases under the trademark Xofigo^©^^1^. Ionic radium is a bone seeking agent, due to its chemical similarity to calcium. It preferentially accumulates in the rapidly forming cells in bone metastases^1, 2^. Radium-223 is the first alpha emitting isotope that obtained FDA approval for the treatment of cancer. Other radium isotopes of interest for preclinical research are ^224^Ra (t_1/2_ 3.6d) and ^225^Ra (t_1/2_ 14.9d). Radium-224 has alpha-therapeutic properties for use similar to that of ^223^Ra, however, it has a shorter half-life. It thus could be more suitable for molecular targets with a shorter biological half-life than that of ^223^Ra^3^ or as a source for the ^212^Pb/^212^Bi generator^4, 5^. Radium-225 is of interest due to its decay to ^225^Ac (t_1/2_ 9.92d), another alpha emitting isotope relevant to the treatment of cancer^6, 7^. There are also non-medical interests in radium isotopes. For instance, ^225^Ra is of interest in the search for an atomic electric-dipole moment^8, 9^.

These radium isotopes are challenging to produce directly, and so are commonly obtained from their radioactive parents’ decay chains. Radium-223 is derived from ^227^Ac (t_1/2_ 21.77y) via ^227^Th (t_1/2_ 18.7d). Actinium-227 comes only from the decay of ^235^U, whose supply is tightly-controlled^10^, or via neutron irradiation of ^226^Ra^11^. Radium-224 forms via the alpha decay of ^228^Th (t_1/2_ 1.91 a). Radium-225 is a decay product of ^229^Th (t_1/2_ 7400 a) and may theoretically be produced by neutron, proton or photon bombardment of radioactive ^226^Ra targets.

Actinium-225 production via spallation of thorium targets is currently being explored at Los Alamos National Laboratory (LANL), Brookhaven National Laboratory (BNL) and Oak Ridge National Laboratory (ORNL)^12^. The amount of ^225^Ac produced by this method represents a small fraction of the total activity produced. Many of the additional radionuclides generated have the potential for use in a variety of medical applications^13–15^. Included in these side products are ^223,224,225^Ra, and the predicted yields of ^223,224,225^Ra along with ^227^Th (parent radionuclide of ^223^Ra) are in the GBq/mAh range^13, 16^. These yields are large enough to justify the investigation of pathways isolating these radium isotopes from the proton irradiated thorium matrix. The separation of radium from thorium has been reported in the literature^17–21^, and in a recent study the recovery of ^223^Ra from proton irradiated thorium target has been described in more detail^22^ In this recent study by Vasiliev *et al*. radium sorption on extraction chromatography “SR resin” has been investigated, and measured HClO_4_ concentration dependent capacity factors for several fission products including radium were reported. The study also presented exemplary SR resin/HNO_3_ and cation exchanger resin/HClO_4_ elution profiles in order to demonstrate radium separation from barium and other fission products. A potential draw-back in this approach is the necessity of an HDEHP/toluene liquid-liquid extraction step for initial bulk thorium removal, which was implemented prior to chromatography-based activation/fission product recovery. Liquid-liquid extraction procedures introduce larger amounts of contaminated solvent waste and complicate remote or automated handling for the purpose of scale-up to routine large-scale radium production. Furthermore, the use of organic solvents like toluene is a complicating factor in the establishment of radium isotopes for human drug applications.

In the present work, we introduce a novel, aqueous recovery process that solely relies on multiple steps of solid-support sorption. For a seamless integration of an additional isotope recovery “module”, we modified our previously developed method for the isolation of ^225^Ac to co-extract radium isotopes (namely ^223,224,225^Ra)^12^. As the production of ^225^Ac remains our main priority, it is important to obtain these radium isotopes in high purity and yield with minimal impact on ^225^Ac recovery. Described within is a method to obtain these radium isotopes in high yield and radiochemical purity (>99.9%) as an ancillary process to the recovery of ^225^Ac.

## Results

### Thorium metal proton irradiation

The thorium target irradiation resulted in the formation of activation products ^223,224,225^Ra along with several fission products. The production yield of ^223,224,225^Ra (10 days post irradiation) in the dissolved 10 g target pre-separation are shown in Table 1. The theoretical cumulative yields for ^223,224,225^Ra (10 days post irradiation), predicted by published cross section measurements^13, 16^, are also shown in Table 1. The discrepancies of the measured values from the predicted values likely stem from the difficulty in integrating gamma peaks with high backgrounds and multiple contributions from various sources, uncertainty in cross-section measurements, and large dilution factors for HPGe measurements^14, 23, 24^.

**Table 1:** Production Yields of ^223, 224, 225^Ra from a 10 g ^232^Th target (90 MeV, 230 µA, 22.5 h, 640 mg/cm^2^).

Isotope	Half-Life (d)	Production Yield (MBq)	Theoretical Cumulative Yield (MBq)
^223^Ra	11.435	1600 ± 100	2600 ± 200^13*^
^224^Ra	3.63	102 ± 21	36 ± 13^16^
^225^Ra	14.9	40 ± 15	42 ± 6^13^

*Includes yield from decay of ^227^Th.

### Chemical Separation

**Figure 1:**
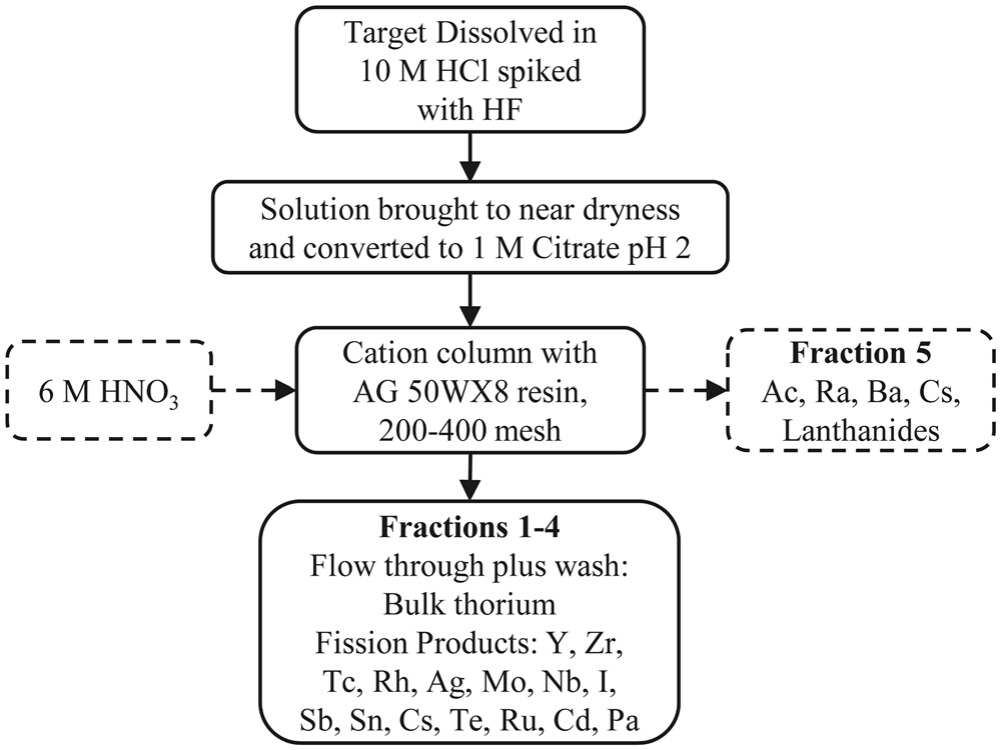
Cation Exchange Chromatography.

**Figure 2:**
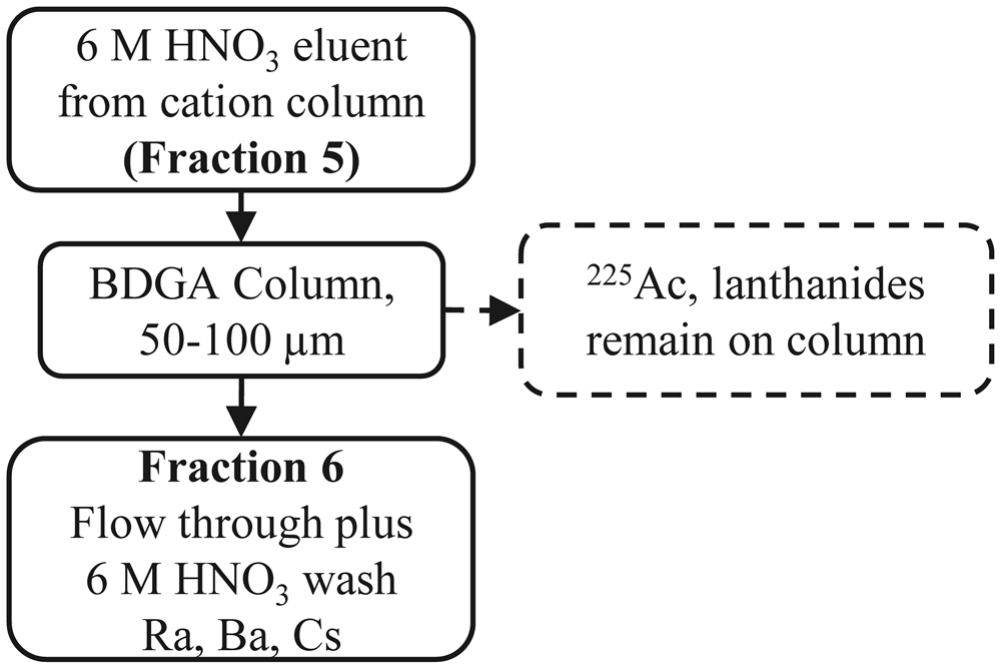
TEHDGA Extraction Chromatography.

**Figure 3:**
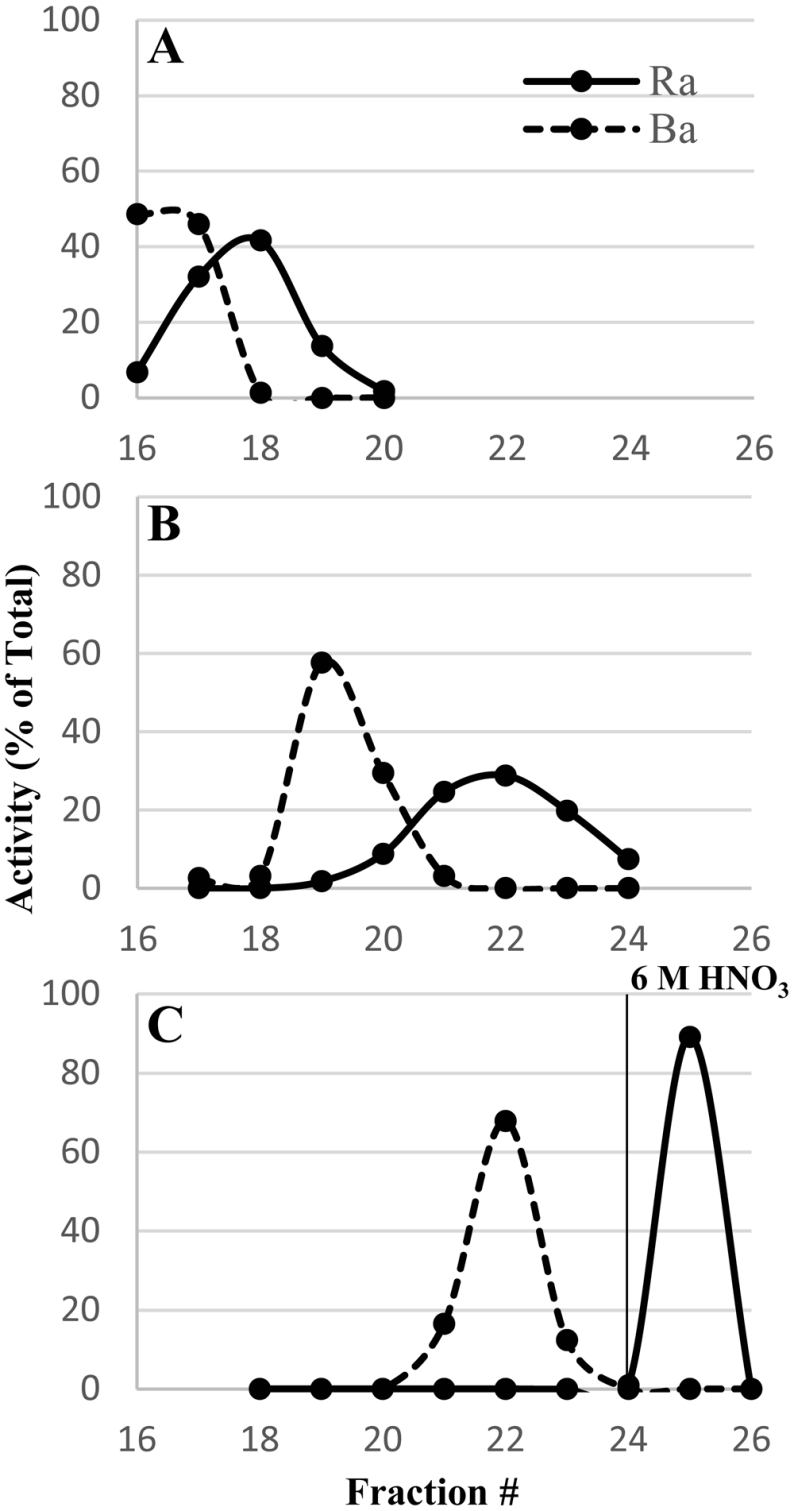
Cation exchange separation of radium/barium separation using 0.32 M Citrate pH 5.5 with (**A**) 1 mL, (**B**) 2 mL, and (**C**) 3 mL of AG 50WX8 resin. In C, radium was eluted with 6 M HNO_3_. Plotted lines are eye guides only.

**Figure 4:**
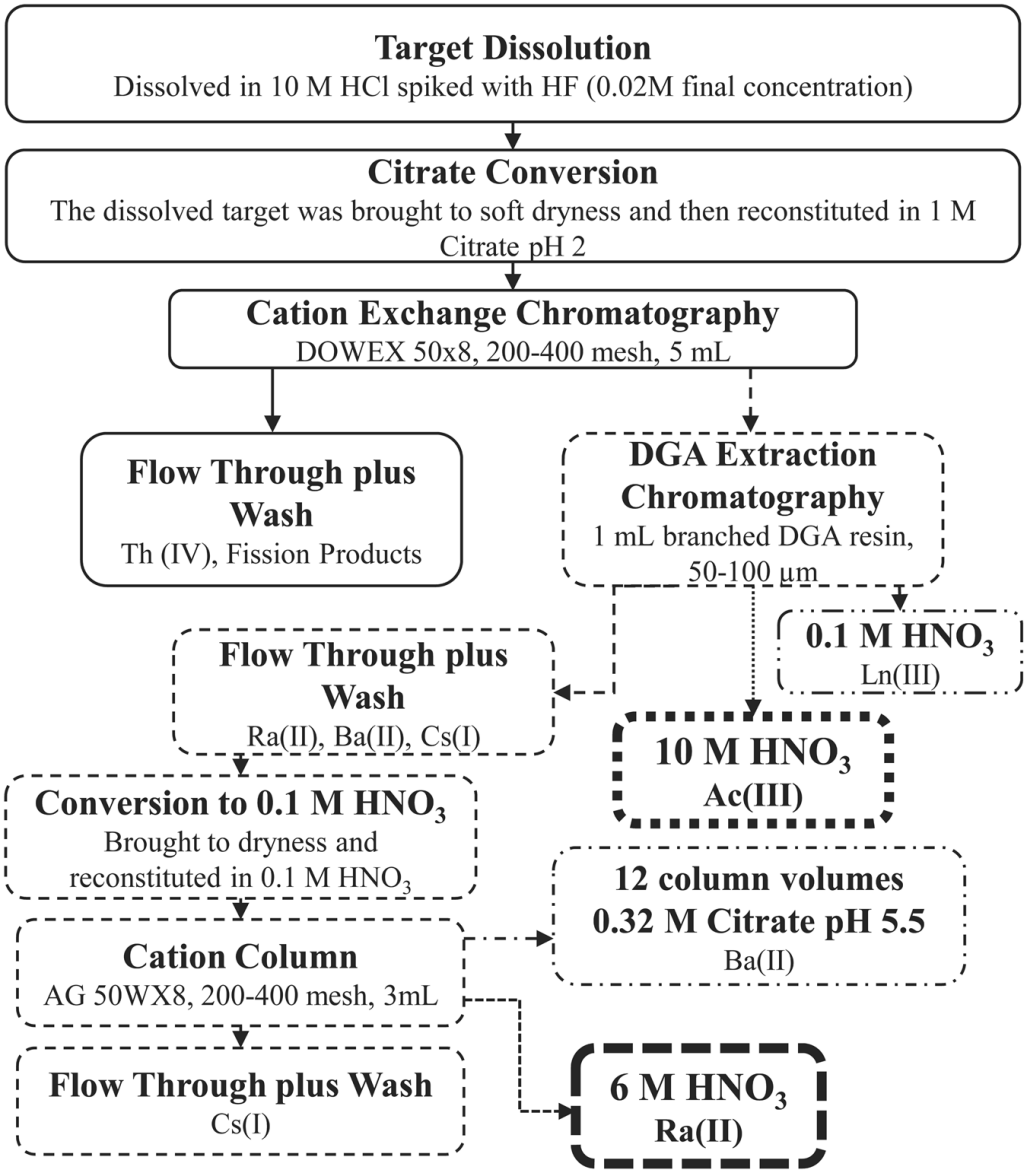
Final ^225^Ac separation schematic with recovery of radium isotopes.

Cation exchange column contact resulted in the separation of the majority of the fission products, fractions 1–4 (Fig. 1). The addition of 6 M nitric acid removed 97 ± 6% of radium along with barium, actinium, cesium, and lanthanides (fraction 5). Loading this fraction onto a TEHDGA) column resulted in the elution of ≥99% of the radium, barium, and cesium isotopes while retaining the remaining isotopes (Fig. 2). Three column bed volumes were chosen to assess Ra/Ba separation behavior in a 0.32 M citrate pH 5.5 mobile phase (or eluent). As shown in Fig. 3, the 1 and 2 mL sized columns resulted only in partial separation of Ra from Ba accompanied by substantial losses. The 3 mL column, however, resulted in an effective separation of radium from barium and a total radium recovery yield of 91 ± 3% with a radiochemical purity of ≥99.9%. Cesium isotopes eluted in the initial loading effluent and also in the 0.1 M HNO_3_ washes. Due to the broad elution peak of radium when using 0.32 M citrate, radium was eluted with 6 M HNO_3_ once barium was no longer detected in the 0.32 M citrate fractions collected from the 3 mL column. The fully integrated separation scheme, as adapted from Radchenko *et al*.^12^ now including the newly developed Ra/Ba separation module, is shown in Fig. 4.

## Discussion

This method uses a three column approach to the separation of radium isotopes from a proton irradiated thorium matrix adding only one additional column to the ^225^Ac separation scheme. The benefit to this process over the previously published process is the elimination of large volumes of radioactive organic wastes^22^. Mixed waste (radioactive organic solutions) are a limitation to many facilities due to the high cost and difficulty of disposal.

One concern for separating radium isotopes from fission products is potential contamination with the strontium isotopes; ^89^Sr and ^90^Sr. These isotopes are of concern due to their long half-lives (50.6 days and 28.8 years respectively) and lack of a strong gamma line for detection. Strontium and barium both demonstrate stronger complexation with citrate than radium^25^. Separation of strontium, barium, and radium with citrate media has been performed by Thompson *et al*. and it is shown that strontium elutes before barium, which in turn elutes before radium^26^. Therefore using this method would effectively remove contaminating strontium isotopes before radium elution.

The importance of supplying radionuclides with high purity and specific activity, especially therapeutic radioisotopes, has been increasingly relevant in the field of nuclear medicine. The results described within show that radium isotopes can be obtained as a by-product of ^225^Ac production with an average radiochemical purity ≥99.9% as determined by activity. Yield estimates establish that quantities up to 0.3 Ci of ^223^Ra and 0.1 Ci of ^225^Ra^13^ and 0.2 Ci of ^224^Ra^16^ can be made and recovered from thorium targets in tandem with anticipated Ci-scale ^225^Ac production at LANL or BNL isotope facilities. Further, the citric acid flow through from the cation exchange column can be reprocessed 21 days after initial purification to obtain ^223^Ra from the decay of ^227^Th. Quantities as high as 4 Ci of ^223^Ra would be available during this second processing^13^. As the radium recovery is performed downstream of the ^225^Ac recovery process, there is no effect on the processing time or yield of ^225^Ac.

Isotopic impurities of radium cannot be separated chemically, however, there are many uses for radium isotopes that do not require isotopic purity. For instance pure ^225^Ac can be obtained from this source after 18 days have passed. Once the ^225^Ac has grown in, separation from the radium isotopes can be obtained employing the TEHDGA column as shown in Fig. 2. Additionally, since ^223^Ra is FDA approved for radiotherapy, there has been interest in developing radium chelates for use in targeted alpha therapy^27^. For the purposes of designing and testing chelates, an isotopically pure form of ^223^Ra would not be necessary. Furthermore, the non-medical interest of ^225^Ra for the measurement of an atomic electric-dipole moment also does not require isotopically pure ^225^Ra. The radium recovered from ^225^Ac processing could provide a consistent supply of radium for these purposes.

## Materials and Methods

### Chemicals

Thorium metal targets were manufactured at the Los Alamos National Laboratory (LANL). Small pieces of thorium metal (purity >99% as determined via X-ray fluorescence spectroscopy) were obtained from LANL’s internal inventory. The raw material was arc melted and rolled into sheets with mean thickness of 0.50 ± 0.02 mm for the use as proton beam targets.

All experiments were performed in triplicate unless stated otherwise. All chemicals were used without further purification. Nitric acid and hydrochloric acid, both Optima grade, were purchased from Fisher Scientific (Pittsburgh, PA, USA). Citric acid (99.9%) was obtained from Sigma–Aldrich (St Louis, MO, USA), and deionized water (≥18 MΩcm) was prepared on site with a Millipore water purification system. Cation exchange resin AG 50WX8, 200–400 mesh, was obtained from Biorad (Hercules, CA, USA). Branched DGA resin (N,N,N′,N′-tetrakis-2-ethylhexyldiglycolamide; abbreviated TEHDGA) 100–150 µm, was purchased from Eichrom Inc. (Lisle, IL, USA).

### Gamma-ray spectrometry

The radioactivity measurements made at ORNL to determine radium yields in proton irradiated targets were conducted using a well-shielded, Canberra Model GC2020 High-Purity Germanium detector with a relative efficiency of 20%. A PC-based multichannel analyzer utilizing Canberra Genie 2000 software was coupled to the detector. The measured resolution of the detector was 2.0 keV at 1.33 MeV. Energy and efficiency calibrations were completed using a γ ray source traceable to the National Institute of Standards and Technology (NIST). Spectra collection times varied from one-hour counts for initial sample dilutions to 36-hour counts for severely decayed samples. Sample to detector geometry was varied to reduce the detector dead time below 5%. Each peak in the γ ray spectra was fitted using the non-linear least squares fit method (Canberra, 2009). When possible, multiple γ ray peaks were used to quantify the activity at end of bombardment (EOB) for each radioisotope through a weighted average method.

For separation experiments gamma-ray spectrometry was conducted using an EG&G Ortec Model GMX-35200-S HPGe detector system in combination with a Canberra Model 35-Plus multichannel analyzer. Detector diameter was 50.0 mm; detector length 53.5 mm; Be window thickness 0.5 mm; outer dead-layer thickness 0.3 µm. Detector response function determination and evaluation were performed using standards of radionuclide mixtures containing ^241^Am, ^109^Cd, ^57^Co, ^139^Ce, ^203^Hg, ^113^Sn, ^137^Cs, ^88^Y, ^60^Co, traceable to the National Institute of Standards and Technology (NIST) and supplied by Eckert & Ziegler, Atlanta, GA, USA. The detector was a p-type Al-windowed HPGe detector with a measured FWHM at 1333 keV of approximately 2.2 keV and a relative efficiency of about 10%. Relative total source activity uncertainties ranged from 2.6% to 3.3%. Counting dead time was kept below 10%.

### Irradiation Conditions

A thorium metal target, 10 g mass, 640 mg/cm^2^ mass thickness, was irradiated at the Isotope Production Facility (IPF), Los Alamos National Laboratory (LANL, NM, USA). The target was encapsulated in Inconel cladding and placed into the high energy “A” slot (90 MeV incident energy) of the IPF target assembly. IPF targetry and 4π water cooling have been described previously^28, 29^. The target was irradiated in a proton beam with average intensity of 230 µA for 22.5 hours. The target was then transported to the IPF Hot Cell Facility where it was removed from the Inconel shell and shipped to Oak Ridge National Laboratory (ORNL) for chemical processing.

### Chemical Separation

#### Cation exchange column

The chemical separation was adapted as follows from previous work in order to extract the radium isotopes with little impact on ^225^Ac recovery^12, 24, 30^. The irradiated 10 g target material was dissolved in 200 mL 10 M HCl and 250 μL of 2 M HF with heating (~70 °C) for approximately 2 hours. Three aliquots of the dissolved target were used to spike three separate solutions of ~1 g of ^232^Th metal dissolved in 40 mL 10 M HCl spiked with 40 μL 2 M HF. Each aliquot represented a mixture of all radionuclides previously identified in the target^12^ and each solution was processed independently according to the following steps. The solution was brought to near dryness and reconstituted in 205 mL of 1 M citrate, pH 2, and passed through a cation exchange column containing 5 mL AG50 × 8 resin, 200–400 mesh, which was preconditioned with a 1 M citrate, pH 2, solution. The eluate from the column was collected, fraction 1, then an additional 50 mL 1 M citrate, pH 2, was used to wash the column, fraction 2. The column was then washed with 10 mL 1 M nitric acid to remove residual citrate, fractions 3 (5 mL) and 4 (5 mL). Twenty-five milliliters of 6 M nitric acid was then added to the column and collected in fraction 5.

### TEHDGA resin

Fraction 5 from each of the replications was then loaded onto separate TEHDGA resin columns, 1 mL 50–100 µm, preconditioned with 6 M nitric acid. The eluate was collected, fraction 6 (25 mL). The column was then washed with an additional 10 mL 6 M nitric acid, fractions 7 (5 mL) and 8 (5 mL). Next, 20 mL of 10 M nitric acid was added to the column and collected, fractions 9–12 (5 mL volume each). Finally, the column was washed with 5 mL 0.1 M nitric acid, fraction 13. All collected fractions were measured using HPGe spectrometry to identify the isotopic and elemental species present.

### Radium/Barium Separation

For the radium/barium separation, a method by Power *et al*. was adapted as described below^31^. Fraction 6–8 from each of the reiterations were brought to dryness, reconstituted in 5 mL 0.1 M HNO_3_ and then passed through cation columns containing 1 mL (0.8 cm inner diameter), 2 mL (0.8 cm inner diameter), or 3 mL (0.8 cm inner diameter) of AG 50WX8 resin, 200–400 mesh, and the effluent was collected, fraction 14 (5 mL). For the 1 mL column, an additional 5 mL of 0.1 M HNO_3_ was added to wash the column, fraction 15, followed by five fractions of 0.32 M citrate pH 5.5, fractions 16–20 (5 mL each). For the 2 mL column, two additional 5 mL washes of 0.1 M HNO_3_ were added to the column and collected to result in fractions 15 (5 mL) and 16 (5 mL), followed by eight 5 mL fractions of 0.32 M citrate pH 5.5 (fractions 17–24, 5 mL each). For the 3 mL volume sized column, three additional 5 mL washes of 0.1 M HNO_3_ were added to the column and collected: fractions 15–17, followed by seven 5 mL fractions of 0.32 M citrate pH 5.5, fractions 18–24. Radium was eluted from the 3 mL column using two 5 mL fractions of 6 M HNO_3_, fractions 25 and 26. All collected fractions were measured using HPGe spectrometry in order to identify the radionuclidic species present.

## Conclusion

A novel, all aqueous methodology for the recovery of Ra isotopes from proton irradiated thorium matrices has been presented in our study. The results presented within show that radium isotopes can be recovered in high yield and radiochemical purity in a process that is ancillary to ^225^Ac production. With the scale-up to 100 g thorium targets, hundreds of millicuries of radium isotopes would be available for recovery. The radium product obtained by this method is composed of ^223^Ra, ^224^Ra, and ^225^Ra and is suitable for chemistry applications (such as chelate development) and the nuclear physics community focused on measurement of the atomic electric-dipole moment. Additionally, the ^225^Ra produced via this method would be suitable as a generator of pure ^225^Ac for clinical applications as the other radium impurities do no decay to actinium isotopes.

### Data Availability

The datasets generated during and/or analyzed during the current study are available from the corresponding author on reasonable request.
